# Film based on magnesium impregnated biochar/cellulose acetate for phosphorus adsorption from aqueous solution†

**DOI:** 10.1039/c8ra06655h

**Published:** 2019-02-14

**Authors:** Marina de Carvalho Eufrásio Pinto, Demetrius David da Silva, Ana Luiza Amorim Gomes, Victor dos Santos Azevedo Leite, Allan Robledo Fialho e Moraes, Roberto Ferreira de Novais, Jairo Tronto, Frederico Garcia Pinto

**Affiliations:** Federal University of Viçosa, Department of Agricultural Engineering, Campus Universitário Viçosa MG CEP 36570-000 Brazil; Federal University of Viçosa, Institute of Exact Science, Campus de Rio Paranaíba Rodovia BR 354 km 310, Rio Paranaíba MG CEP 38810-000 Brazil fredericogarcia.ufv@gmail.com; Federal University of Viçosa, Institute of Agrarian Science, Campus de Rio Paranaíba Rodovia BR 354 km 310, Rio Paranaíba MG CEP 38810-000 Brazil

## Abstract

Phosphorus (P) is a nutrient necessary for agricultural production and a potential originator for eutrophication in water bodies, resulting in qualitative changes; it may also affect the aquatic ecosystem and human health. In addition, as a finite resource, the importance of studying strategies to remove it from water is evident, thus making possible its recycling. Many studies have used powdered materials, including biochars, for P water decontamination; however, the difficulty of separating and collecting these materials from water after adsorption may be difficult. Therefore, using hybrid materials in which the fine particles (powder) are impregnated into larger, solid particles by means of a polymeric host can facilitate collection and reuse after P adsorption. In this context, this study aimed the synthesis and characterization of a new hybrid film formed by the biopolymer cellulose acetate (CA) and biochar (FAC-B) for P adsorption in aqueous solution. We obtained biochar from the pyrolysis of carrot residue (*Daucus carota* L.) and doped it with magnesium. As a biodegradable polymer and the most abundant natural polysaccharide in the environment, using CA as a biochar support material is an environmentally friendly alternative. We prepared the CA film with the casting method, and the biochar was inserted into the filmogenic solution in the same amount as the CA. The film was characterized by X-ray diffraction (XRD), thermogravimetric analysis (TGA), differential scanning calorimetry (DSC), molecular absorption spectroscopy in the infrared region with an attenuated total reflectance (FTIR/ATR) accessory, and X-ray Photoelectron Spectroscopy (XPS). We evaluated the thickness, weight, density, H_2_O uptake and H_2_O solubility of the produced FAC-B. The maximum adsorption capacity of P by FAC-B was 21.57 mg g^−1^, in agreement with the Langmuir isotherm model. The adsorption value suggests that the film has the potential to be used as an efficient P adsorbent in water.

## Abstract 2

New hybrid film formed by biopolymer cellulose acetate and biochar for P adsorption in aqueous solution.

## Graphical abstract

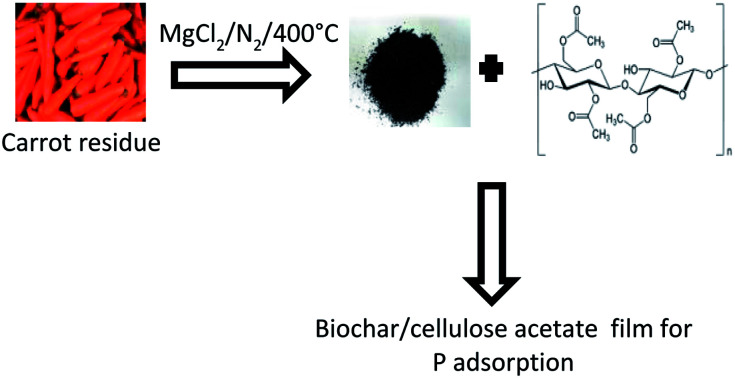

## 1. Introduction

Biochars are materials produced by thermal degradation of biomass under limited O_2_ (pyrolysis) conditions,^1^ which have been widely applied as adsorbents of phosphorus (P) in aqueous solution.^2,3^ Increasing phosphorus concentration in water bodies can cause eutrophication, leading to changes such as in pH, dissolved oxygen and water transparency, which may affect the aquatic ecosystem and human health.^4^

P is a finite and essential element for plant development and, with the continuous increase of the world population, greater quantities of this element are necessary to guarantee food production.^5,6^ Moreover, in tropical soils, much of the P applied is not available to the plants, being occluded in the soil retained by minerals such as gibbsite and goethite, causing large quantities of phosphate fertilizers to be applied to achieve high crop yields.^7^ Thus, strongly fixed in the soil, P can be dragged to bodies of water by surface runoff and erosion, causing contamination.^8–10^

The adsorption of P in a biochar without any additional treatment is unlikely due to the presence of carboxylic and phenolic groups that prevent the adsorption of anions such as those from P.^11–13^

Different techniques for the chemical modification of the biochar have been used for the insertion of cations in the biochars, allowing to reach high rates of P removal due to the appearance of a cation bridge between the biochar and the anion to be adsorbed.^13–19^ In this work, was performed the doping of the biochar using magnesium.

Recent studies have shown that magnesium-modified (Mg) biochars are effective at removing P in aqueous solution.^12,20–24^ However, even with a great capacity for P removal in aqueous solution, the difficulty of collecting and reusing powdered materials after the adsorption process can make the use of the biochars as adsorbent materials less attractive.^25^ According to Shepherd *et al.*,^26^ it is of great importance that the captured P is subsequently recycled, rather than becoming a waste product of the process. An effective alternative to solving this problem is the synthesis of hybrid composites by impregnating or coating fine particles into larger sized, solid particles^27^ using a polymeric host.^28^

Biodegradable biopolymers are an environmentally friendly alternative, since they come from renewable and biodegradable sources. Composites using biodegradable biopolymers as matrices to support different adsorbents materials have been studied for contaminant removal in aqueous solution. Among them are spherical composites using cellulose matrices,^29,30^ calcium alginate,^31,32^ chitosan,^33,34^ and still, fibrous composites of chitosan (Razzaz *et al.*, 2016).^35^

Cellulose acetate (CA) is a biodegradable thermoplastic polymer derived from cellulose esterification, which is the most abundant natural polysaccharide in the environment.^36^ Depending on its degree of substitution (GS), CA is used in the manufacture of different commercial products, such as fabrics, plastics, photographic films and cigarette filters.^37^

In a large research carried out in the specialized literature, there were no reports on biochar immobilization in biodegradable CA films. Thus, this work aimed at synthesizing a hybrid film of cellulose acetate and biochar from carrot residue pretreated with magnesium, its characterization and, furthermore, adsorption studies of P in this new material.

## 2. Experimental

Milli-Q® deionized H_2_O was used in this study to prepare all the solutions, and also to wash the carrot to obtain the biochar. All used chemical reagents have analytical purity. The reagents were KH_2_PO_4_ Dinâmica 99%, MgCl_2_·6H_2_O Vetec 99%, NaOH neon 98.4%, HCl Moderna 37%, acetone P.A., chromate and cellulose acetate (AC), Rhodia. N_2_ gas used in biochar synthesis was from White Martins at 99.99% purity.

### 2.1. Biochar from pre-treated carrot in magnesium solution

For biochar synthesis, was used carrot residue (*Daucus carota* L.). The carrot is an interesting raw material for the production of biochar as it is one of the most efficient crops in the accumulation of biomass.^38^ In Brazil, high yields of carrot production have been achieved in crops (above 80 t ha^−1^).^39^ However, according to reports from producers 20 to 40% of supplied carrot is discarded for different reasons, generating large quantities of organic waste. Thus, it is interesting to study a reuse of this waste biomass. The carrot sample was collected in a crop in the region of Alto Paranaíba, state of Minas Gerais, Brazil. The carrot sample was washed, processed and oven dried at 80 °C for 72 h. The dried biomass was then ground in a knife mill and sieved to a particle size of less than 30 mesh. This ground material was immersed in a solution containing 120 g MgCl_2_·6H_2_O dissolved in 360 mL deionized H_2_O, with mass ratio of carrot to solution volume of 1 : 10 and kept under magnetic stirring for 2 h. Subsequently, the biomass treated in magnesium solution was dried in an oven at 80 °C for 72 h and ground them in the knife mill again. The powder was then pyrolyzed at 400 °C for 2 h in a tubular oven under N_2_ atmosphere. After this procedure, the biochars were macerated and sieved through a 100 mesh.

### 2.2. Biodegradable cellulose acetate film

The cellulose acetate film was prepared with the casting method.^40^ In order to obtain the filmogenic solution, we solubilized CA in acetone at the ratio of 1 : 10 (m/v), resting for 24 h in a completely sealed glass vial at room temperature. Then, the solution was subjected to magnetic stirring for 2 h. The filmogenic solution was poured onto a glass plate, remaining at rest until complete solvent evaporation. The cellulose acetate film was named FAC.

### 2.3. Biodegradable film of cellulose acetate and biochar

CA was solubilized in acetone as described previously. After the resting period of 24 h, the solution was subjected to magnetic stirring and the biochar was slowly added with a ratio of CA to biochar of 1 : 1 (m m^−1^). After 2 h of stirring, the resulting filmogenic solution was applied onto the glass plate for solvent evaporation, resulting in cellulose acetate and biochar (FAC-B) film.

All materials synthesized in this study were stored in a vacuum desiccator with silica gel until the time of its use.

Images of the filmogenic solution during FAC-B preparation and of this same film ready to be used in the adsorption tests are presented as ESI, corresponding to Fig. S1 and S2,† respectively.

### 2.4. Characterization of materials

#### 2.4.1. X-rays diffraction (XRD)

XRD analysis of biochar samples, FAC, FAC-B, and the cellulose acetate film with biochar after P adsorption (FAC-B-P) occurred using a machinery Shimadzu XRD-6000 with a graphite crystal as a monochromator to select the Cu-Kα1 radiation with *λ* = 1.5406 Å and a step of 0.02° s^−1^, with a 2*θ* scanning angle between 4 and 70°.

#### 2.4.2. Thermogravimetric analysis (TGA)

Thermogravimetric analysis took place on equipment DTG 60H (Shimadzu Co., Japan). We heated about 3.0 mg biochar, FAC and FAC-B from 25 °C to 700 °C at a heating rate of 10 °C min^−1^ under N_2_ atmosphere (50 mL min^−1^).^41^ The decomposition temperatures of the compounds were obtained from the first derivative of mass loss (%) *versus* temperature (DTGA).

#### 2.4.3. Differential scanning calorimetry (DSC)

Differential scanning calorimetry analysis of FAC and FAC-B occurred by using equipment DSC 60 (Shimadzu Co., Japan). We submitted 7.0 mg samples to the following conditions: heating from 30 °C to 255 °C at a heating rate of 10 °C min^−1^. The N_2_ flow was 50 mL min^−1^.

#### 2.4.4. Molecular absorption spectroscopy in the infrared region with attenuated total reflectance (FTIR/ATR)

The FTIR/ATR analysis was for biochar, FAC, and FAC-B on the Spectrometer Jasco, model FT/IR-4100. The spectra were obtained with a resolution of 4 cm^−1^ in a wavelength range from 4000 to 400 cm^−1^ with 256 scannings for each spectrum.

#### 2.4.5. X-ray photoelectron spectroscopy (XPS)

XPS measurements were performed in a UNI-SPECS UHV spectrometer. The spectra were obtained using a magnesium source with Kα line (*E* = 1253.6 eV) and the energy pass through the analyzer was set to 10 eV. The pressure in the analytical chamber was less than 10^−7^ Pa. The binding energy scale was calibrated using line 4f of Au. The surface composition of the material was evaluated based on the binding sites and energies of the central levels C 1s, O 1s, Mg 1s and P 2p.

#### 2.4.6. Thickness, weight and density of films

FAC and FAC-B thicknesses were measured at 5 random points and the mean values were calculated. For this, we used a digital micrometer (produced by Mitutoyo Corporation, Japan) model ID-C112XB, with a 0.001 mm resolution. To obtain the film weights, the dry sample masses was divided by their areas, and calculated the film densities by the ratio of the masses of the film samples to the volumes thereof.

#### 2.4.7. Water absorption in films

The degree of swelling of FAC and FAC-B was determined in agreement to the method of Jipa *et al.* slightly modified using eqn (1).^42^1
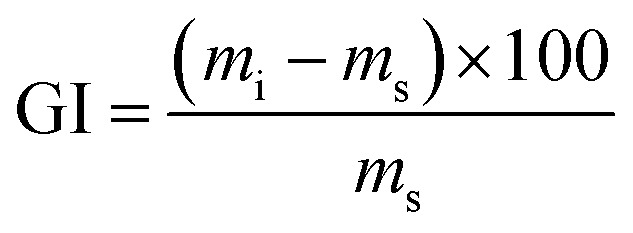
in which GI is the degree of swelling of the film (%), *m*_i_ is the mass of the swollen film (g) and *m*_s_ is the mass of the dry film (g).

To obtain the swollen mass, we submerged film samples measuring 7 × 2.5 cm in 50 mL H_2_O for 2 h at 25 °C. After this period, excess H_2_O from film surface was removed using an absorbent paper and the masses of the swollen samples were obtained in triplicate and the mean values were obtained for FAC and FAC-B.

#### 2.4.8. Solubility in water (*S*_H_2_O_)

For estimate of FAC-B solubility in H_2_O, FAC-B samples (7 × 2.5 cm) were submerged in 50 mL vials containing H_2_O (in triplicate) and kept under stirring at 25 °C. After shaking for 48 h, the film samples were dried and weighed. Eqn (2) calculated *S*_H_2_O_.2
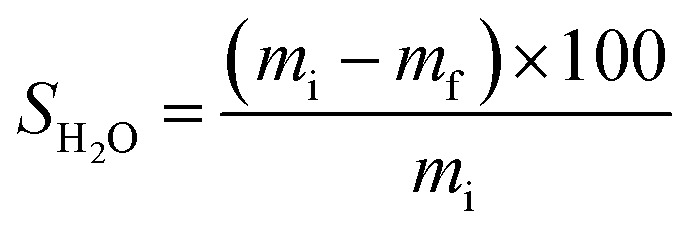
in which *S*_H_2_O_ is the solubility in H_2_O of the film (%), *m*_i_ is the film initial mass (g) and *m*_f_ is the film mass at the end of 48 hours of stirring in H_2_O (g).

### 2.5. Experiments of adsorption of P in aqueous solution

An aqueous stock solution of 1000 mg L^−1^ of P was prepared by dissolving KH_2_PO_4_ in H_2_O and, thereafter, we diluted this solution at different concentrations to conduct the adsorption experiments.

P adsorption experiments in the films were in triplicate and the mean values reported. The molybdenum blue spectrophotometric method determined P concentrations in solution in a UV-vis spectrophotometer (Thermo model Evolution 300).^43^

Using eqn (3), the amount of adsorbed P was calculated.3
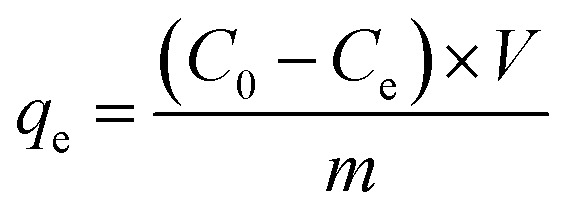
in which *q*_e_ is the amount of P adsorbed (mg g^−1^), *C*_0_ is the initial concentration (mg L^−1^), *C*_e_ is the balance concentration (mg L^−1^), *V* is the solvent volume (L), and *m* is the mass of adsorbent sample (g).

#### 2.5.1. Kinetics of adsorption

In the kinetics of adsorption study, FAC-B with a mass of around 2.5 g were kept under stirring in bottles containing 500 mL P solution (50 mg L^−1^) in a thermostated bath at 25 °C. At different time intervals, ranging from 0.5 h to 96 h, samples were withdrawn from the suspensions and P concentrations were determined. We used the kinetics models of pseudo-first order (eqn (4)) and pseudo-second order (eqn (5)) to simulate experimental kinetics.4
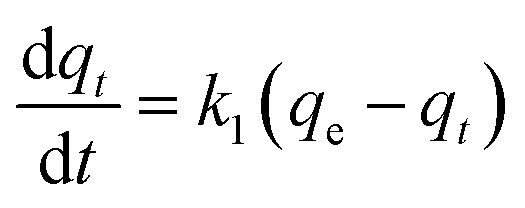
5
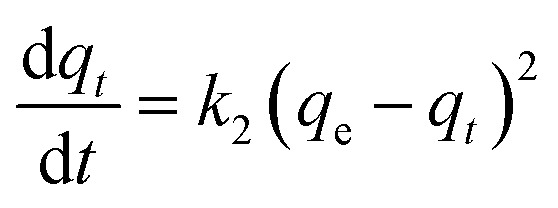
in which, *k*_1_ and *k*_2_ are respectively the adsorption rate constants of pseudo-first order and pseudo-second order (h^−1^), *q*_e_ and *q*_*t*_ are the adsorbed amounts per gram of adsorbent at equilibrium and in time *t* respectively (mg g^−1^).

#### 2.5.2. Isotherm of adsorption

To construct the adsorption isotherms, films of size 7.0 × 2.5 cm (around 250 mg) of FAC-B were immersed in 50 mL solutions of P, at different concentrations, ranging from 2.5 to 200 mg L^−1^. The suspensions remained under stirring in a thermostated bath at 25 °C for 48 h. The Langmuir (eqn (6)) and Freundlich (eqn (7)) isotherms models were used to describe P adsorption in FAC-B.6
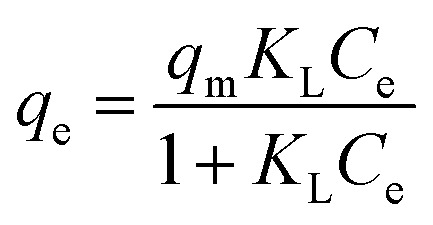
7
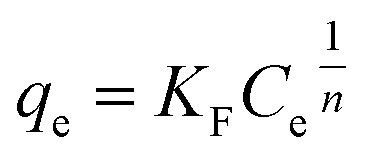
in which, *q*_e_ is the adsorbed amount of solute at equilibrium per gram of adsorbent (mg g^−1^), *C*_e_ is the concentration of solute in equilibrium (mg L^−1^), *q*_m_ (mg g^−1^) is the maximum adsorption capacity, *K*_L_ and *K*_F_ are respectively the Langmuir and Freundlich constants and *n* is a constant that reports adsorption intensity.

#### 2.5.3. Influence of pH value on P adsorption

To evaluate the pH effect of the initial solution on P adsorption, FAC-B of 7.0 × 2.5 cm dimensions (approximately 250 mg) were added to 50 mL solutions of P with a concentration of 85 mg L^−1^ at different pH values (2, 4, 6, 8 and 10) and kept under stirring at 25 °C for 48 h. To adjust the pH values, were used HCl and NaOH 0.1 mol L^−1^ solutions.

## 3. Results and discussion

### 3.1. Characterization of materials

#### 3.1.1. X-rays diffraction (XRD)

**Fig. 1:**
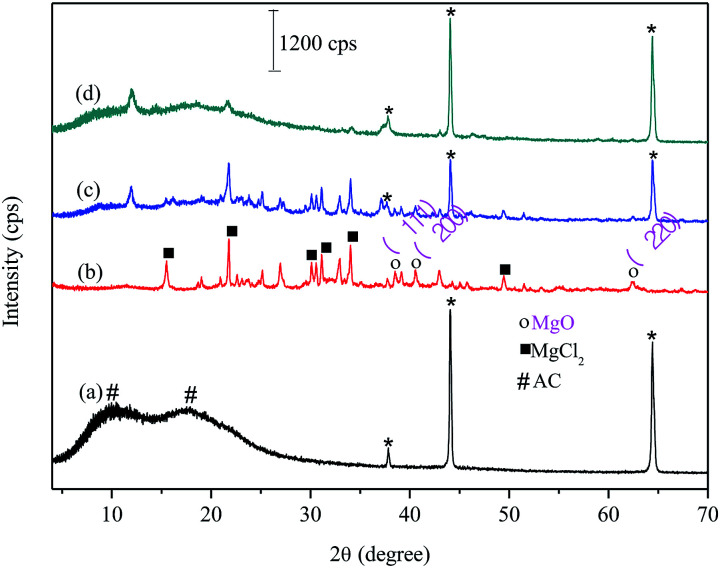
X-ray diffractograms for (a) cellulose acetate film (FAC); (b) biochar; (c) cellulose acetate/biochar film (FAC-B); (d) cellulose acetate/biochar film after P adsorption (FAC-B-P). * = peaks referring to the aluminium sample holder.

Fig. 1 shows X-ray diffractograms for biochar from carrot residues and pretreated with Mg, FAC, FAC-B and FAC-B-P.

For FAC (Fig. 1a), the diffractogram showed two extremely broad peaks and low intensity 2*θ* between 8 and 25°. This diffraction pattern refers to the onset of a disorder in the material due to cellulose acetylation (#). The biochar XRD (Fig. 1b) showed diffraction peaks related to MgCl_2_ (■) from the doping method of biochar and peaks related to the MgO (○) formation in the material. For FAC-B, the diffractogram shows characteristics of a material containing the mixture of polymer and biochar (Fig. 1c). The diffractogram presents the profile of peaks referring to the biochar and low intensity peaks related to CA presence. In relation to FAC-B after P adsorption (Fig. 1d), several peaks referring to biochar disappeared. XPS analyzes were performed in the FAC-B demonstrating the formation of the Mg(H_2_PO_4_)_2_ after the adsorption of P in the film. In addition, the concentration of magnesium in the FAC-B after the adsorption of P was determined and it decreased by 25%. Thus, most of the magnesium still remained impregnated in the polymer matrix after adsorption of P. The decrease of peak intensities displayed in Fig. 1d is probably due to the formation of species with low crystallinity on the surface of the material and the leaching of part of the magnesium during the adsorption process. The peaks relative to CA remained unchanged showing material stability after P adsorption.

#### 3.1.2. Thermogravimetric analysis (TGA)

**Fig. 2:**
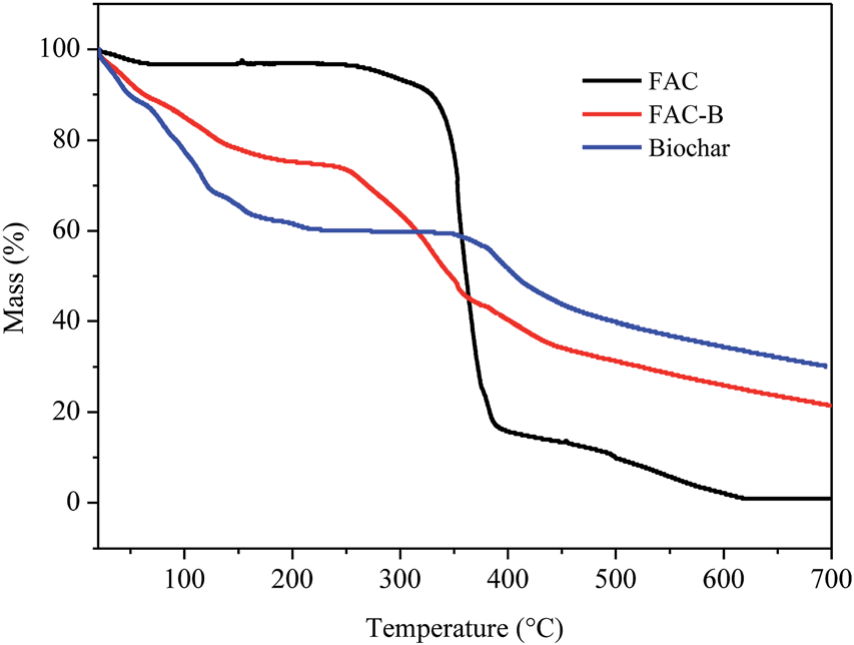
Thermogravimetric analysis (TGA) for cellulose acetate film (FAC), cellulose acetate/biochar film (FAC-B) and biochar.

Fig. 2 shows the TGA curves for FAC, biochar and FAC-B.

According to the TGA thermogram obtained for the FAC, initially, there is a slight mass loss of 3% up to 200 °C, corresponding to the loss of volatile compounds and H_2_O bound to the hydrophilic (OH) groups of the CA chains and, subsequently, to CA deacetylation.^44–48^

There were two more stages of thermal decomposition for this material; the first step was between 300 and 400 °C, with mass loss of 79%, and the second was between 400 and 600 °C, with mass loss of 14%. The first stage (300–400 °C) corresponds to the main thermal decomposition event and can be attributed to CA chain degradation due to the breakdown of glycosidic bonds followed by the primary decomposition in volatile and dehydrated compounds. The final stage of mass loss (400–600 °C) is attributed to sample carbonization, resulting in complete degradation and decomposition of the film.^44–48^

For biochar, were observed two stages of mass loss. The first stage was between 30 and 230 °C, with mass loss of 40% which, according to Zhang *et al.*^20^ can be attributed to the loss of H_2_O adsorbed on the material and by the degradation of lignocellulosic fractions did not decompose during the pyrolysis process at 400 °C. The second stage of biochar thermal decomposition, starting at 380 °C, can be attributed to the release of minerals and salts from the material as also observed by Cimò *et al.*^49^ The final mass of this material corresponded to approximately 35% of its initial mass, indicating its high mineral residue content.

From the thermogram of FAC-B, we observed three stages of thermal decomposition between the ranges 30–200 °C, 215–380 °C, and above 380 °C. This thermogram showed an intermediate profile in relation to the thermograms of FAC and biochar, that is, for each of the temperature ranges of the cited thermal events, their mass variation occurred approximately as an average of the other two thermograms, because the film is formed by 50% by weight of each component. The first step, with mass loss of approximately 20%, can be attributed mainly to the release of H_2_O by the material by the presence of biochar, with the FAC mass in this temperature range practically constant. The second decomposition step is probably due to degradation of CA chain, with the biochar mass remaining almost unchanged. FAC mass loss in this stage was 80%. The third and final step can be attributed to polymer carbonization with total film degradation and part of biochar. FAC-B showed thermal stability lower than the FAC with maximum mass loss of CA at 335 and 360 °C, respectively. The onset temperatures for this event also followed the same behaviour for the two materials, 314 °C for FAC and 230 °C for FAC-B. This phenomenon, in its turn, means that the FAC functioned merely as a physical support for the biochar which is quite positive since the active sites of biochar remains available for phosphorus adsorption from aqueous solution.

#### 3.1.3. Differential scanning calorimetry (DSC)

**Fig. 3:**
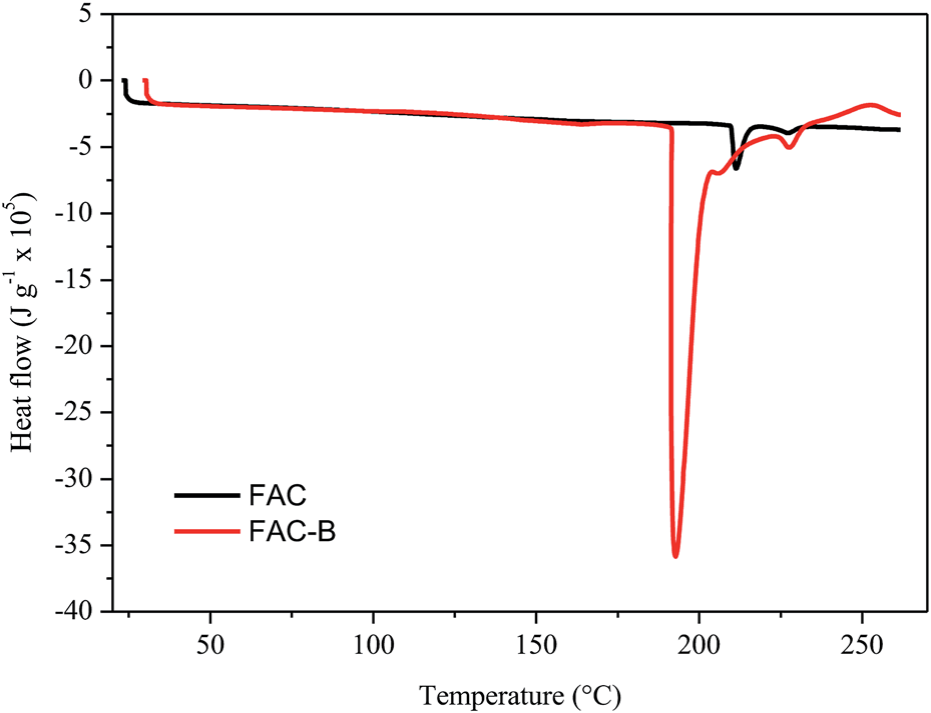
Differential scanning calorimetry (DSC) for cellulose acetate film (FAC) and cellulose acetate/biochar film (FAC-B).

Fig. 3 shows the DSC thermograms of FAC and FAC-B.

The DSC thermograms obtained for FAC and FAC-B showed similar profiles; the main difference was the initial temperature, peak and final melting temperature, as well as the enthalpy involved in this process. The initial, peak and final temperatures for FAC were 210, 211 and 216 °C, respectively, whereas for FAC-B were 190, 192 and 204 °C, respectively. FAC-B melting occurred at a temperature lower than FAC melting. This fact may be attributed to a weakening as well as a smaller number of interactions between the CA chains due to the presence of the biochar in the film. The melting enthalpy for FAC was 660 kJ g^−1^ while for FAC-B it was 3600 kJ g^−1^. The higher energy involved during the FAC-B melting process may be due to water volatilization, since TGA showed large mass loss in this temperature range.

#### 3.1.4. Molecular absorption spectroscopy in the infrared region with attenuated total reflectance (FTIR/ATR)

**Fig. 4:**
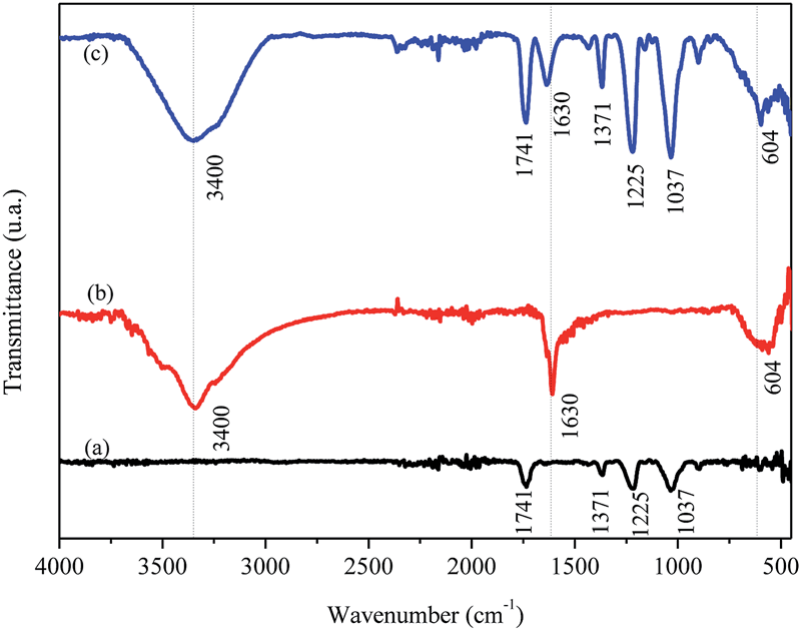
Molecular absorption spectroscopy in the infrared region with attenuated total reflectance (FTIR/ATR) for the (a) cellulose acetate film (FAC), (b) biochar and (c) cellulose acetate/biochar film (FAC-B).

Fig. 4 shows the FTIR/ATR spectra for FAC, biochar and FAC-B.

The FTIR/ATR spectrum for FAC (Fig. 4a) shows a band at 1741 cm^−1^, typical of the formation of CA chains, attributed to the vibrational stretching of carbonyl (C=O) groups of esters present in the CA molecule. There were also other characteristic bands for this material, among them two, one stand out in 1037 cm^−1^ related to vibrational modes of the C–O–C binding and another in 1225 cm^−1^ by stretching the C–O binding, both present in the CA molecule. A small band was observed at 1371 cm^−1^ which may associate with the stretching of the C–H binding of –CH_3_ groups present on acetate radicals. For the biochar (Fig. 4b), a band centered at 3400 cm^−1^ appeared, attributed to the stretching of O–H bidings of hydroxyl groups and molecules of H_2_O in this material. There were another two bands in the biochar spectrum, one in 1630 cm^−1^ characteristic of carbonaceous materials related to the stretching of C=C bindings of aromatics^50^ or by stretching the C=O binding of carboxylates and ketones^14^ and the other in 604 cm^−1^ attributed to Mg–O vibrations in the biochar.^51^ The same bands observed in FAC and in biochar appeared in FAC-B, which was expected because the film was formed by 50% mass of both materials.

#### 3.1.5. X-ray photoelectron spectroscopy (XPS)

**Fig. 5:**
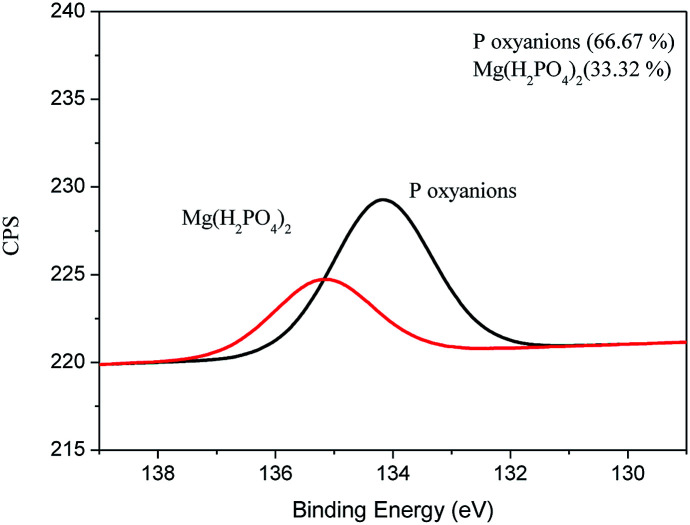
Phosphorus XPS spectrum of FAC-B after phosphorus adsorption.

XPS analysis of the film was performed after the adsorption of P. For the adsorption of P on the surface of the film were placed 2.5 g of film in 500 mL of solution of P (50 mg L^−1^) for a period of 36 h. Fig. 5 shows the XPS spectrum for element P.

It can be seen from Fig. 5 that Mg(H_2_PO_4_)_2_ was formed on the surface of the film after adsorption of P, probably due to the reaction between the oxyanions of P and the MgO present in the material. In addition, it was possible to observe the presence of oxides of P on the surface of the material probably connected by electrostatic attraction on the surface of the film. The XPS spectra for C, O, and Mg can be found in the supplemental material (Fig. S3†). Similar result was obtained by Yao *et al.* (2013) using engineered biochar prepared from Mg-enriched tomato tissues.

#### 3.1.6. Thickness, weight, density, water absorption and water solubility of FAC and FAC-B films

Table S1† shows the results obtained for thickness, weight, density, degree of swelling and water solubility of FAC and FAC-B.

Table S1† shows the FAC thickness modified after the incorporation of biochar in the film. The FAC-B hybrid material had higher film thickness. These variations in thickness may also influence other properties of these materials, such as H_2_O uptake.^52^

From the data shown in Table S1,† there was a large difference in GI (water absorption) between FAC and FAC-B. This H_2_O uptake depends on the hydrophilicity and chemical nature of the materials,^46^ also on its morphological structure.^53^ Thus, a higher GI occurred for FAC-B compared to FAC, which can be attributed to the greater porosity and thickness of FAC-B, as well as to hydrophilic character of the biochar in the hybrid film.

As biodegradable films are originally used for food packaging or encapsulation, water solubility is a desirable feature.^42^ However, in other applications, as evaluated in this study, water insolubility improves material integrity and water resistance.^42,54^Table 1 shows that, after 48 h of FAC-B contact with water, 33% of the film mass was solubilized in water.

**Table 1:** Comparison of adsorption capacity of several polymeric adsorbents for P removal

Adsorbent	Adsorption capacity (mg g^−1^)	References
Gel derived from orange residue treated with Ca(OH)_2_	13.94	55
Wood fiber	4.30	56
Coconut fiber doped with zinc	5.10	57
Collagen fiber loaded with zirconium	28.47	58
Polyurethane foam	11.78	59
Refined aspen wood fiber	4.3	60
Fe_3_O_4_/polysulfone ultrafiltration membrane	0.684	61
Anion-functionalized nanoporous polymer	4.92	62
Hybrid film of cellulose acetate and biochar made from carrot residue	21.57	This work

### 3.2. P adsorption

#### 3.2.1. Kinetics of adsorption

**Fig. 6:**
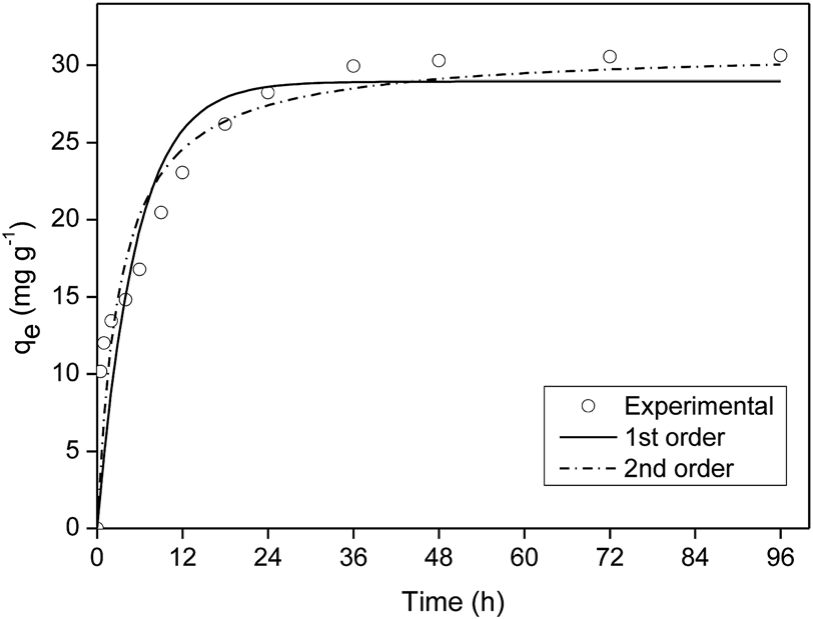
Kinetics of phosphorus adsorption onto cellulose acetate/biochar film (FAC-B).

Fig. 6 shows that after 24 h of contact of FAC-B film with solution containing P, 74.4% of the *q*_e_ value was reached; balance occurred in approximately 48 h. According to Yao *et al.*,^12^ the slow kinetics suggests that precipitation has no important role in P removal by biochar.

The kinetics model of pseudo-first order had the best fit for the experimental data with coefficient of determination (*R*^2^) equal to 0.98 (Table S2†).

#### 3.2.2. Isotherm of adsorption

**Fig. 7:**
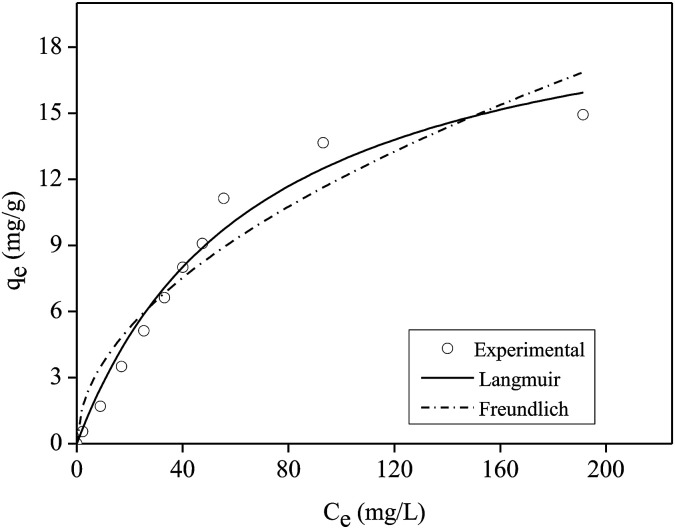
Isotherm of phosphorus adsorption onto cellulose acetate/biochar film (FAC-B).

Fig. 7 shows the experimental data of isotherm of P adsorption in FAC-B, as well as the adjustment to the Langmuir and Freundlich models. The Langmuir model was better fitted to the experimental data, with *R*^2^ = 0.97 (Table S3†). Thus, P adsorption in FAC-B probably occurs as monolayer on a homogeneous surface. P maximum adsorption capacity obtained by the Langmuir equation was 21.57 mg g^−1^.

Table 1 shows the maximum adsorption capacity values of P in aqueous solution obtained by the Langmuir model in other studies that used films or other polymeric materials.

According to Table 1, the film shown in this work had high efficiency when compared to other polymeric materials presented in the literature that were applied for P adsorption in aqueous solution.

#### 3.2.3. pH influence

**Fig. 8:**
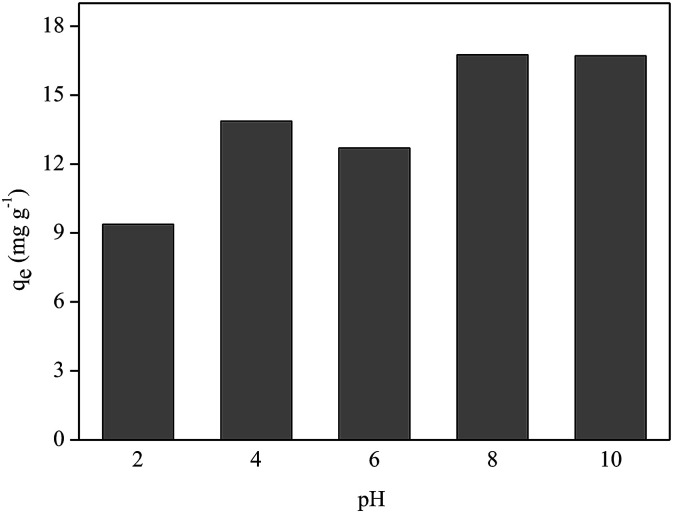
Effect of pH on phosphorus adsorption onto cellulose acetate/biochar film (FAC-B).

The effect of varying the pH value of the medium on P adsorption by FAC-B has been studied and Fig. 8 shows the results.

Fig. 8 shows that better P adsorption results by FAC-B occurred at basic pH values. Despite the existence of OH^−^ anions in the medium at basic pH, the competition between these anions and the oxyanions of P (HPO_4_^2−^; PO_4_^3−^) by the adsorptive sites of the film was not sufficient to overcome the advantage of having P oxyanions in the more deprotonated forms at these pH values to bind to the film, which has a positive surface by the presence of biochar doped with magnesium. At pH values = 2, 4 and 6, even though there are no competing anions in the medium, P species are in the most protonated forms (H_3_PO_4_, H_2_PO_4_^−^), which diminished the interaction of these species with the film surface.

#### 3.2.4. Co-exist anions influence

**Fig. 9:**
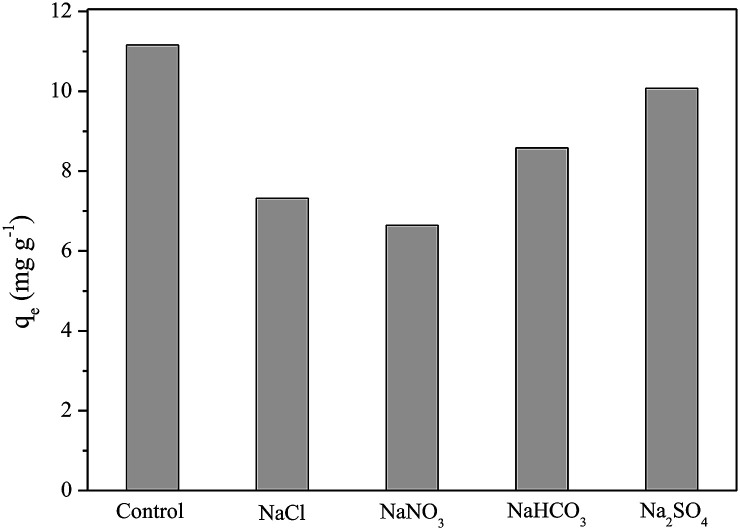
Effect of coexisting anions on phosphorus adsorption onto cellulose acetate/biochar film (FAC-B).

It was observed from Fig. 9 that the adsorption capacity of P decreases when coexisting ions are present in solution, however, the film still exhibits good performance and affinity for P even with the other anions in solution.

## 4. Conclusions

The cellulose acetate and biochar hybrid film made from carrot residue pretreated with magnesium (FAC-B) showed high efficiency when compared to other polymeric materials from the literature, applied to P adsorption in aqueous solution.

P maximum adsorption capacity by FAC-B was 21.57 mg g^−1^, according to the Langmuir isotherm model and kinetics of adsorption was the pseudo-first order.

The influence study of pH value of the medium on P adsorption by FAC-B showed that better results are for pH values 8 and 10.

The developed hybrid film is easy to use in adsorption studies, with advantages in relation to adsorbent materials in the form of powder, such as the non-agglomeration of adsorbent particles and their ease of removal from the medium for possible adsorbent recycling, with great potential for environmental applications.

## Conflicts of interest

There are no conflicts to declare.

## Supplementary Material

RA-009-C8RA06655H-s001
