# Supplementary material for: Film based on magnesium impregnated biochar/cellulose acetate for phosphorus adsorption from aqueous solution
Source: RSC Adv. 2019 Feb 14;9(10):5620–7. doi: 10.1039/c8ra06655h (PMC9060768; doi:10.1039/c8ra06655h)
Supplement: RA-009-C8RA06655H-s001 [file RA-009-C8RA06655H-s001.pdf]

Electronic Supplementary Material (ESI) for RSC Advances.
This journal is © The Royal Society of Chemistry 2019

SUPPLEMENTARY MATERIAL

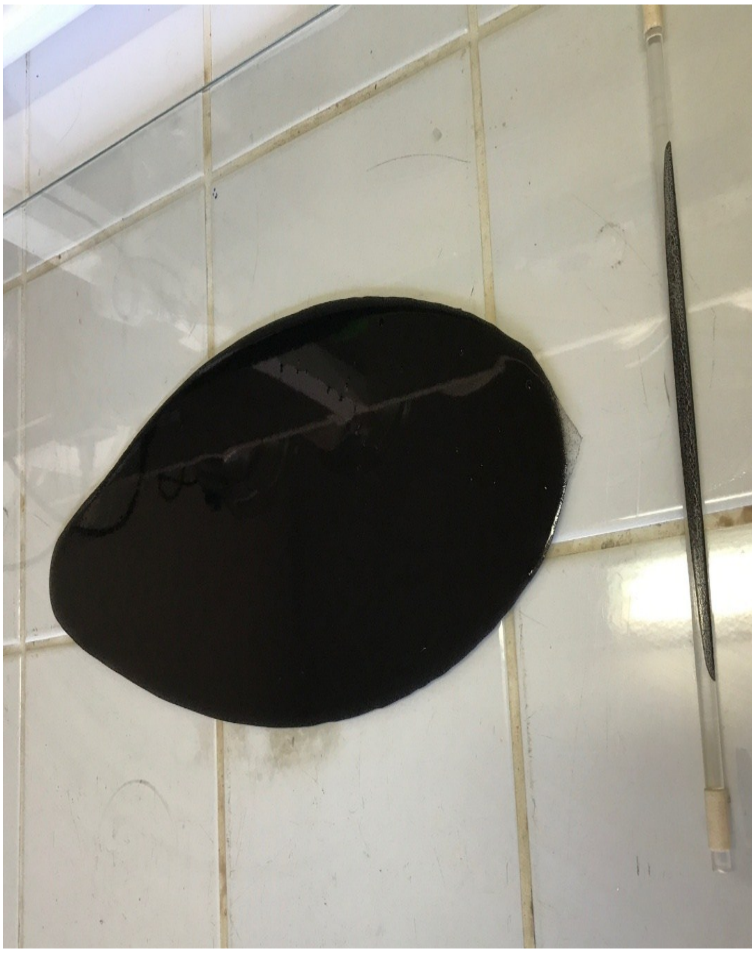

Fig. S1 Filmogenic solution of cellulose acetate and biochar applied on glass, in acetone drying process.

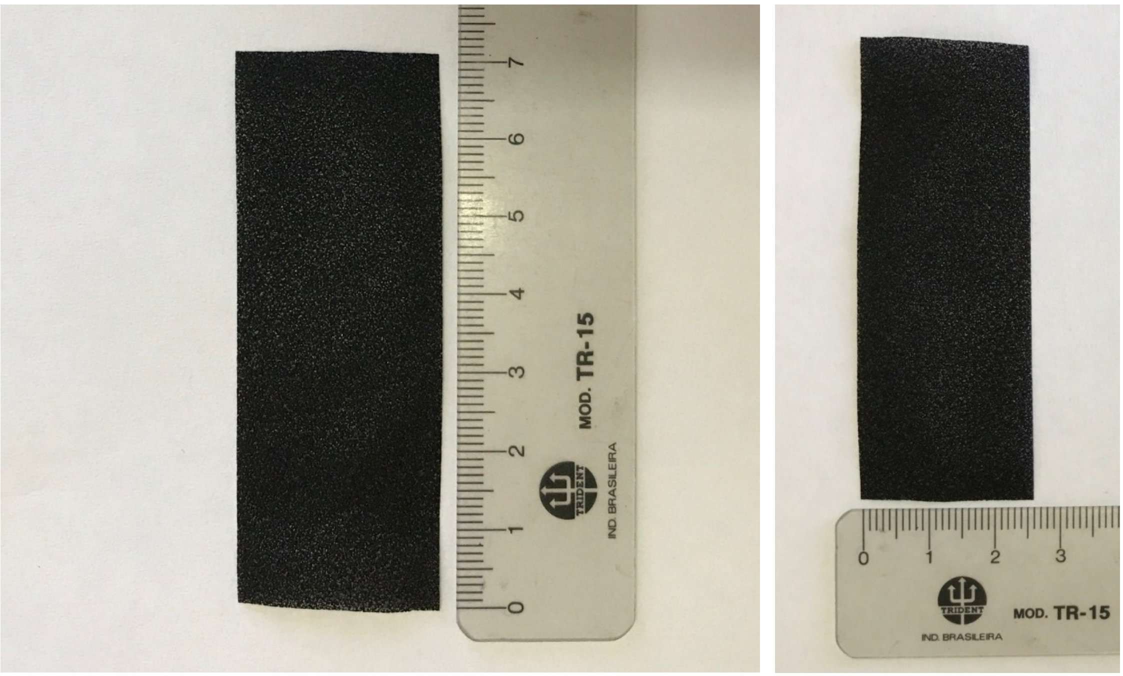

Fig. S2 Hybrid film of cellulose acetate and dried biochar and cut to size 7.0 x 2.5 cm, for use in adsorption studies.

Table S1 Thickness, weight, density, degree of swelling (*GI*), and solubility in FAC and FAC-B H_2O (S_{H2O})

Film	Thickness (µm)	Weight (g m^{-2})	Density (g cm^{-3})	GI (%)	S_{H2O} (%)
FAC	43.4 ± 2.3	53.3 ± 1.7	1.2 ± 0.1	9.1 ± 1.8	1.4 ± 0.1
FAC-B	221.0 ± 12.3	143.1 ± 7.3	0.6 ± 0.1	27.7 ± 1.8	33.8 ± 0.9

Table S2 Kinetics of P adsorption parameters

Model	Parameter 1	Parameter 2	R^2
First order	k_1 = 0.05 ± 0.01 (h^{-1})	q_e = 7.19 ± 0.29 (mg g^{-1})	0.98
Second order	k_2 = 0.01 ± 0.01 (g mg^{-1} h^{-1})	q_e =9.30 ± 0.76 (mg g^{-1})	0.97

Table S3 Isotherm parameters of P adsorption in the FAC-B

Model	Parameter 1	Parameter 2	R^2
Langmuir	k_l = 0.02 ± 0.01 (L mg^{-1})	q_e = 21.57 ± 1.92 (mg g^{-1})	0.97
Freundlich	k_f = 1.13 ± 0.34 (mg$^{(1-n)}$ L g^{-1})	n = 1.94 ± 0.26	0.91

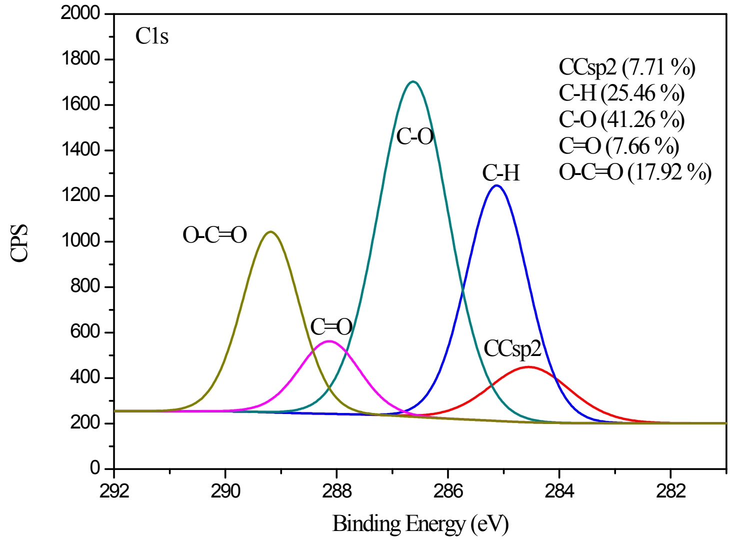
C1s
CCsp2 (7.71 %)
C-H (25.46 %)
C-O (41.26 %)
C=O (7.66 %)
O-C=O (17.92 %)
O-C=O
C=O
C-O
C-H
CCsp2
CPS
Binding Energy (eV)

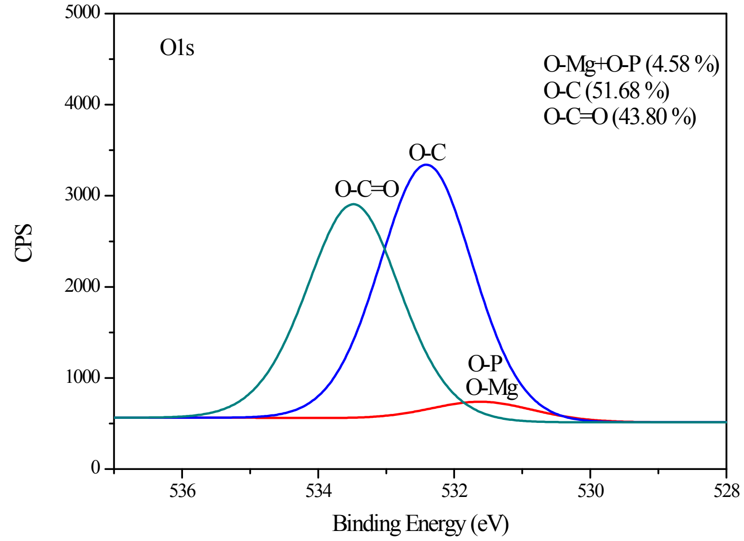
O1s
O-Mg+O-P (4.58 %)
O-C (51.68 %)
O-C=O (43.80 %)
O-C=O
O-C
O-P
O-Mg
CPS
Binding Energy (eV)

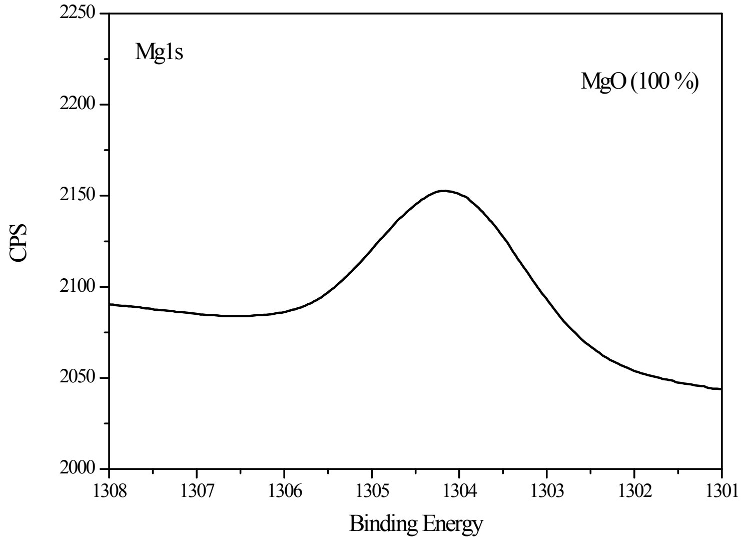

Fig. S3 Carbon, Oxygen, and Magnesium XPS spectrum of FAC-B after phosphorus adsorption.
